# Clinical Significance and Potential Mechanisms of the RNA Methyltransferase KIAA1429 in Osteosarcoma

**DOI:** 10.7150/jca.86630

**Published:** 2024-01-01

**Authors:** Yu Sun, Yi-wu Lei, Jia-xing Zeng, Lu-yang Zhong, Jian-wei Liu, Yu-nan Man, Mao-lin He

**Affiliations:** 1 Division of Spinal Surgery, The First Affiliated Hospital of Guangxi Medical University, Shuangyong Road 6, Nanning 530021, Guangxi Zhuang Autonomous Region, China.; 2 Department of Radiology, The First Affiliated Hospital, Guangxi Medical University, Shuangyong Road 6, Nanning 530021, Guangxi Zhuang Autonomous Region, China.; 3 Trauma Microsurgical Hand Surgery, Guangxi Zhuang Autonomous Region People's Hospital, Taoyuan Road 6, Nanning 530021, Guangxi Zhuang Autonomous Region, China.; 4 Department of Osteology, The Second People's Hospital of Nanning, The Third Affiliated Hospital of Guangxi Medical University, Dancun Road 13, Nanning 530031, Guangxi, China.

**Keywords:** osteosarcoma, RNA methyltransferase, KIAA1429, oncogene.

## Abstract

**Background:** KIAA1429, a member of the RNA methyltransferase complex, is involved in cancer progression; however, the clinical significance and underlying mechanism of KIAA1429 in osteosarcoma (OS) remains to be reported.

**Methods:** We evaluated the clinical significance of KIAA1429 in OS by performing RT-qPCR, microarray, and RNA sequencing and using published data as a reference. Two KIAA1429-targeting siRNA constructs were transfected into SW1353 cells. CCK-8 assay, colony formation assays, flow cytometry and the xenograft mouse model were conducted to investigate the biological function of KIAA1429 in OS.

**Results:** The mRNA expression of KIAA1429 was markedly upregulated in 250 OS samples as compared to that in 71 non-cancer samples (standardized mean difference = 0.67). Summary receiver operating characteristic curve analysis revealed that KIAA1429 exhibited reliable diagnostic capacity to differentiate OS samples from non-cancer samples (area under the curve = 0.83**)**. Further, survival analysis indicated that KIAA1429 overexpression was associated with shorter overall survival time. Knocking down KIAA1429 reduced m6A methylation levels, inhibited proliferation, prevented the growth of tumors *in vivo* and accelerated apoptosis of OS cells. In total, 395 KIAA1429-related genes were identified among co-expressed genes and differentially expressed genes, which were enriched in the cell cycle pathway. Protein-protein interaction network analysis showed that CDK1, CCNA2, and CCNB1 were KIAA1429-related genes, serving as major network hubs in OS.

**Conclusions:** Our findings indicate that KIAA1429 plays an oncogenic role in OS and potentially facilitates OS progression via a mechanism that involves regulating CDK1, CCNA2, and CCNB1.

## Introduction

Osteosarcoma (OS) is the most common primary malignant bone tumor derived from mesenchymal tissue, and accounts for approximately 35% of primary malignant bone tumors 1. OS occurs predominantly in the metaphysis of long bones, and the average annual incidence of OS is about 4 cases per million 2. Meanwhile, OS occurs mainly in adolescents and is the third deadliest tumor among adolescents 3. Despite advances in neoadjuvant chemotherapy and surgical techniques, OS generally remains insensitive to common adjuvant treatment methods, such as radiotherapy and chemotherapy 4, 5. So far, nearly 40 years, the survival rate of patients with OS has not improved 6. Therefore, there is an urgent need to better understand the molecular mechanisms of OS development and to identify novel therapeutic targets.

In eukaryotes, N6-methyladenosine (m6A) is the commonest post-transcriptional modification of RNA 7. M6A modification is mainly impacted by three types of proteins, including methyltransferase, demethylase, and m6A binding protein 8. Proteins that modify or regulate m6A can accelerate the proliferation, migration, and metastasis of malignant tumor cells. For example, METTL3 that reported as a m6A “writer” was shown to post-transcriptionally silence SOCS2 expression through a YTHDF2-dependent pathway to promote liver cancer progression 9. Ma *et al.* found that METTL14, a m6A “writer”, affected the metastasis of liver cancer by mediating the maturation of miRNA-126 10. FTO as a member of m6A “eraser”, Zhou *et al.* reported that the FTO expression levels were notably increased in cervical squamous cell carcinoma, and elevated FTO was associated with poorer clinical prognosis 11. Additionally, FTO promoted progression of leukemia by reducing ASB2 and RARA m6A levels 12. The m6A demethylase ALKBH5 was found to maintain the tumorigenicity of glioma stem cells by regulating FOXM1 expression 13, while, ALKBH5 promotes OS progression by upregulating PVT1. Recent studies have shown that METTL3 promotes OS growth by upregulating ATAD2 through m6A methylation modification 14. Furthermore, METTL3 functions as an oncogene in OS by upregulating DRG1 and promoting progression of OS 15.

KIAA1429 is a subunit component of the m6A methyltransferase complex, and participates in the modification of m6A residues in RNA 16. In human A549 cells, deletion of KIAA1429 significantly reduced the maximum levels of m6A in RNA, indicating that KIAA1429 plays an important role in mediating m6A methyltransferase activity 17. KIAA1429 has also been shown to participate in tumor progression through both m6A-dependent and m6A-independent mechanisms 18-21. In OS, only one study reported that KIAA1429 is regulated by miR-143-3p and promotes OS progression 22. However, the clinical significance of the KIAA1429 RNA methyltransferase in OS and the potential mechanisms by which it contributes to OS progression remain incompletely understood.

In present study, we comprehensively assessed the clinical significance of RNA methyltransferase KIAA1429 and investigated its biological role in OS. Our research shows that KIAA1429 is significantly increased in OS and plays an oncogenic role in the progression of OS. Furthermore, functional enrichment analysis identified a potential biological mechanism through which KIAA1429 contributes to OS.

Thus, our study aims to explore the mechanism of KIAA1429 in OS, and put forward the hypothesis that KIAA1429 may promote the progress of OS by targeting CDK1, CCNA2, and CCNB2, hoping to provide new insights for exploring the potential biomarkers and therapeutic targets of OS. A flow chart outlining this study is shown in Supplementary Fig. 1.

## Materials and Methods

### Clinical samples

This study involved 30 patients (16 men and 14 women) with OS (osteosarcoma) who were hospitalized during 2014 to 2019 for surgical treatment at the First Affiliated Hospital of Guangxi Medical University and none of them had received chemotherapy or radiotherapy (as shown in Supplementary table 1). The age range of patients was 9-31 years (median age, 14 years): three were < 10 years old, 22 were 10-20 years old, and five were > 20 years old. OS lesions were located in the lower femur, upper tibia, and upper fibula in 18, 7, and 5 patients, respectively. We collected OS tissues and their adjacent tissues from each patient. The inclusion criteria were as follows: (1) clear clinical diagnosis of OS; (2) site of tumor onset should have been regions such as the large joints of long bones; and (3) tumor should not have metastasized before surgical treatment.

### Ethical approval

Informed consent was obtained from all patients. This study was approved by the Ethics Committee of the First Affiliated Hospital of Guangxi Medical University.

### Data collection

OS gene expression data were retrieved from the following databases: Gene Expression Omnibus (GEO), ArrayExpress, and Sequence Read Archive (SRA). The search terms used were: (bone OR bones) OR (osteosarcoma OR osteosarcomas). The data inclusion criteria were: (1) human samples; (2) the study included an OS group and a non-cancer group; (3) the study included KIAA1429 expression data. The data exclusion criteria were: (1) radiotherapy, chemotherapy, or targeted therapy; (2) distant metastasis; (3) uncertain diagnosis or mixed tumor.

We obtained KIAA1429 expression data for OS samples and corresponding clinical data from the Therapeutically Applicable Research to Generate Effective Treatments (TARGET) database. In addition, microarray, RNA sequencing, and published studies including KIAA1429 expression and prognostic clinical data were used to assess the prognostic value of KIAA1429 in OS.

### Cell culture and transfection

Human OS cell lines MG-63, Saos2, and SW1353, and osteoblast cell line hFOB1.19 were obtained from the Cell Bank of the Chinese Academy of Science (Shanghai, China). Cells were cultured in Roswell Park Memorial Institute-1640 medium containing 10% fetal bovine serum (Gibco) and 1% penicillin/streptomycin (Solarbio) in humidified air (containing 5% CO_2_) at 37°C. Two si-KIAA1429 constructs (si-KIAA1429-1, CAGUGAUGUUCAAAUGCUAGA; si-KIAA1429-2, GGAAGAACCAAGACUACUAAA) and a negative control siRNA construct (si-NC) were synthesized by Shanghai Genechem Company Ltd. SW1353 cells were infected with lentiviral particles, and the infected cells were selected with 3 μg/ml puromycin 23.

### RT-qPCR

Total RNA was extracted using TRIzol (Invitrogen, USA). Synthesis of cDNA was performed using a One-Step RT-PCR Kit (Thermo Fisher, USA). Real-time PCR assay was performed using an ABI Vii7 system (Applied Biosystems, USA). GAPDH mRNA expression was used as a reference gene. The primers synthesized were: KIAA1429-hF GTTGTGCCACCACCAAGAGG and KIAA1429-hR AACCCACCACGGGAAGAAAT; GAPDH-hF TGACAACTTTGGTATCGTGGAAGG and GAPDH-hR AGGCAGGGATGATGTTCTGGAGAG. Relative expression data was calculated using the 2^-ΔΔCt^ method 24. To explain, the CT value refers to the number of amplification cycles required for the fluorescence signal of an amplicon to reach a set fluorescence threshold during RT-qPCR. In other words, the Ct value represents the fractional cycle number in the log-linear region of PCR amplification in which the reaction reaches a fixed amount of amplicon DNA. Differences in gene expression levels can be measured when a statistically significant increase in fluorescence is first detected. In this study, the Ct value of the reference gene GAPDH was subtracted from that of the target gene KIAA1429 for each group (normal and OS tissues) to derive ΔCt1 and ΔCt2. After subtracting the ΔCt2 value from the ΔCt1 value, the negative reciprocal of the result represented the 2^-ΔΔCt^ value. The 2^-ΔΔCt^ value indicated the expression fold of KIAA1429 in OS tissues relative to that in normal tissues.

### Western blot protein analysis

Western blotting was performed as described 25 with antibodies against KIAA1429 and GAPDH (Santa Cruz Biotechnology, USA); the latter was used as an endogenous control to normalize expression values of KIAA1429.

### M6A RNA methylation quantification

EpiQuik m6A RNA Methylation Quantification Kit (Colorimetric) was used to assess m6A methylation levels, as previously described 26.

### Cell proliferation assay and colony formation assays

Cell counting kit-8 assay (CCK-8, Beyotime, Shanghai, China) was used to measure the cell proliferation. Briefly, SW1353 cells (5×10^3^ cells/well) were seeded into 96-well plates, incubated overnight, and treated with control, si-NC, si-KIAA1429-1, or si-KIAA1429-2 for 0, 24, 48, 72, and 92 h. Then, cells were incubated with CCK-8 reagent (10 μL) for 2 h at 37°C and the absorbance was measured at 450 nm 27. SW1353 cells were added to a six-well plate to test the development of colonies. Then, as previously explained, cultures were fixed, stained, and cell counts were performed 28.

### Apoptosis assay

An Annexin V-FITC Apoptosis Detection Kit (Becton Dickinson San Jose, CA) was used. After treatment, 5×10^5^cells were pelleted by centrifugation, resuspended in binding buffer (200 μl), and incubated with 5 μl fluorescein isothiocyanate-Annexin V and 1 μl propidium iodide (PI) solution for 30 min at room temperature. Cells were detected using a Calibur Flow Cytometer (BD); apoptotic cells stained positive for Annexin V and negative for PI 29.

### Xenograft tumor model

BALB/c nude mice, aged four to six weeks, were procured from the Shanghai Institute of Materia Medica, Chinese Academy of Science, and kept under specific pathogen-free conditions. The experiments were performed as previously described 30, and they were reviewed, approved, and supervised by the First Affiliated Hospital of Guangxi Medical University's Ethics Committee, the ethical approval number is 2023-E438-01.

### Identification of KIAA1429-related genes

KIAA1429-related genes were identified from co-expressed genes (CEGs) and differentially expressed genes (DEGs) in OS. To define genes as CEGs, we estimated the relationships between expression of genes and KIAA1429 expression in the microarray and RNA sequencing datasets; we considered genes with |Pearson's r| ≥ 0.3 and *p* < 0.05 and appear in at least three datasets as KIAA1429 CEGs. Differentially expressed genes (DEGs) were defined using the limma package in R software, based on microarray and RNA sequencing datasets 31, 32. Specifically, for microarray datasets, we chose the limma package; the voom method in the limma package was used to analyze count data from RNA-seq. Besides, we converted FPKM value to TPM value using this transformation formula: 
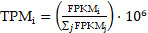
. Subsequently, DEGs were identified via the limma package. *p* < 0.05 and |log_2_FC| > 1 were the screening criteria. Genes that appeared in at least three datasets were selected as DEGs. Genes fitting within the criteria in both screens from the CEGs and the DEGs were considered to be KIAA1429-related genes.

### Bioinformatics analysis

The Database for Annotation, Visualization, and Integrated Discovery (DAVID) database was used to perform enrichment analysis for Gene Ontology (GO) functions and Kyoto Encyclopedia of Genes and Genomes (KEGG) pathways enriched in the KIAA1429-related genes 33. The Search Tool for the Retrieval of Interacting Genes (STRING) database and Cytoscape software were used to construct the protein-protein interaction (PPI) network of KIAA1429-related genes 34, 35. Hub related genes were screened based on the degree of connectivity among KIAA1429-related genes.

### Validation of hub related genes

Firstly, the expression of hub related genes in OS was evaluated by analyzing microarray and mRNA sequencing data. Secondly, the diagnostic potential of hub related genes was evaluated by a summarized receiver operating characteristic (SROC) curve. Finally, the prognostic value of hub related genes was analyzed based on the microarray, RNA sequencing, and published data.

### Statistical analysis

Independent t-tests were employed to assess the expression differences of KIAA1429 between two independent groups. Receiver operating characteristic (ROC) curves were applied to determine the sensitivity and specificity of KIAA1429 in each study. Stata12.0 was applied to merge standardized mean difference (SMD), summary receiver operating characteristic (SROC), hazard radio (HR) and its 95% confidence interval (95% CI). Cochran's Q test and I^2^ index were employed to evaluate the heterogeneity between the included studies. If *p* < 0.05 or I^2^ > 50%, there was significant heterogeneity between the included studies, and a random-effects model was applied; otherwise, a fixed-effects model was used. Sensitivity analysis was employed to assess the robustness of each study included in the meta-analysis. Begg's test and Egger's test were used to evaluate whether included studies exist publication bias. A p-value of* p* < 0.05 was considered to be statistically significant.

## Results

### Expression of KIAA1429 in OS

**Figure 1:**
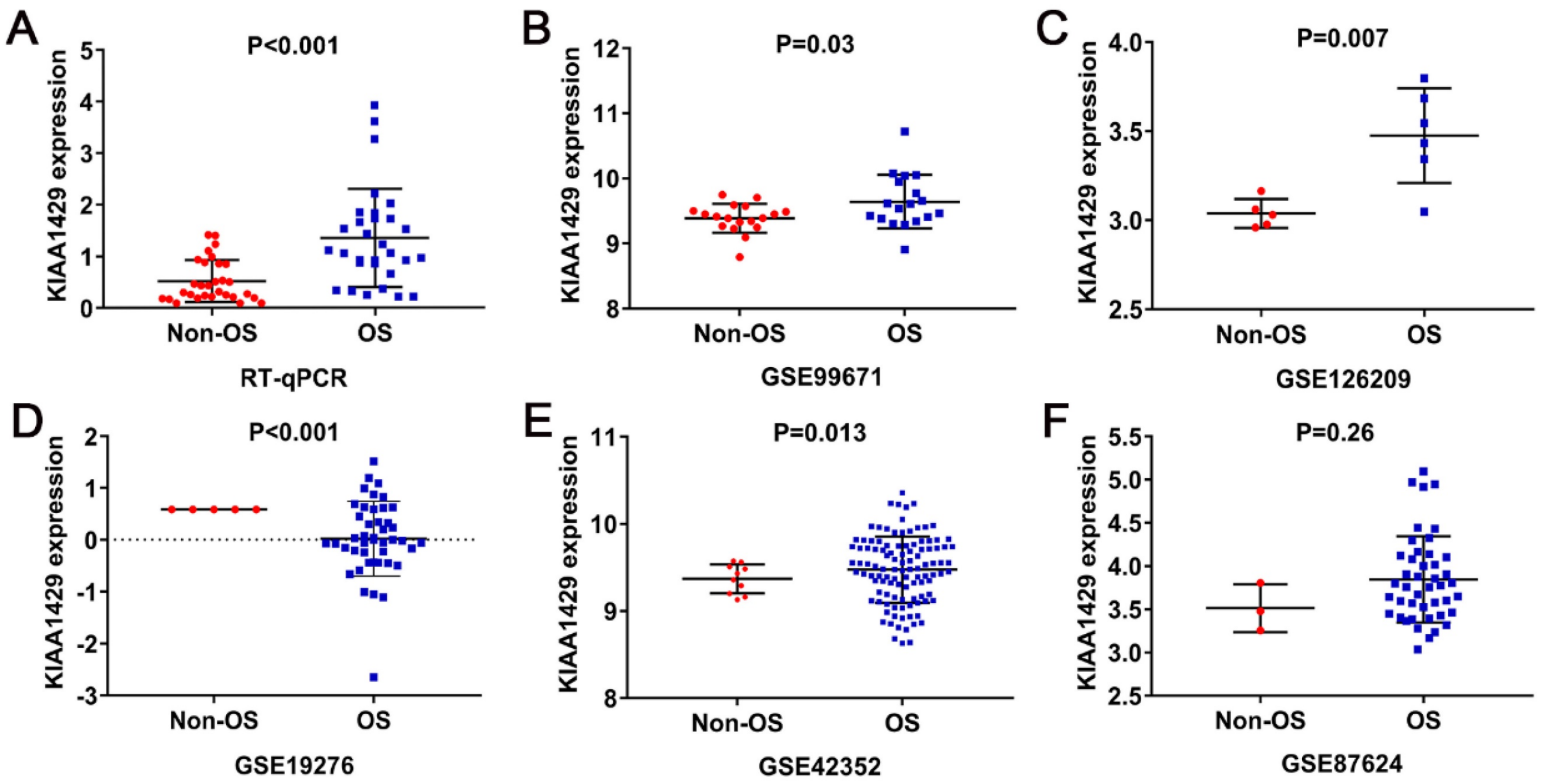
** The expression level of KIAA1429 in OS based on RT-qPCR, microarray and RNA sequencing data.** (A) RT-qPCR. (B) GSE99671. (C) GSE126209. (D) GSE19276. (E) GSE42352. (F) GSE87624. OS: osteosarcoma.

**Figure 2:**
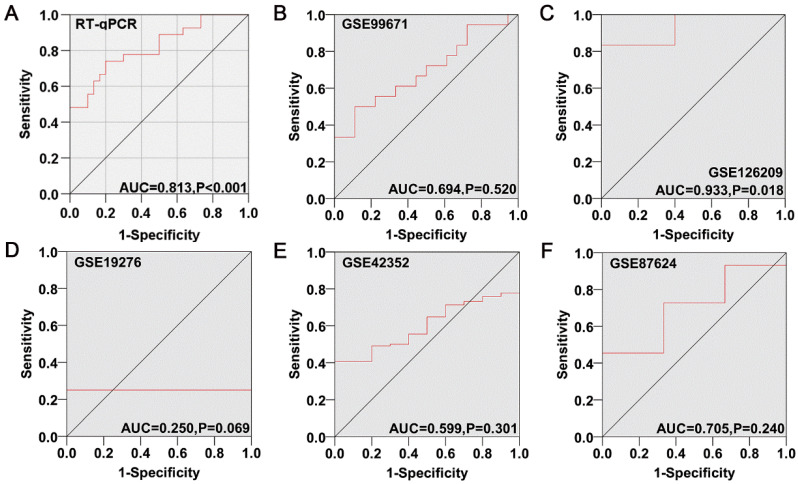
** Diagnostic capability of KIAA1429 in OS based on RT-qPCR, microarray and RNA sequencing data.** (A) RT-qPCR. (B) GSE99671. (C) GSE126209. (D) GSE19276. (E) GSE42352. (F) GSE87624. OS: osteosarcoma.

KIAA1429 expression was significantly higher in 30 OS clinical samples than in 30 non-cancer clinical samples, as indicated by RT-qPCR (Fig. 1A). Five studies met the inclusion criteria for analyses. In three datasets (GSE99671, GSE126209, and GSE42352), KIAA1429 was significantly overexpressed in OS samples; in one study (GSE19276), KIAA1429 expression was significantly lower in OS samples; and in the final study (GSE87624), there was no notable difference in KIAA1429 expression between OS and non-cancer samples (Fig. 1B-F). The potential of KIAA1429 to distinguish OS samples from non-cancer samples in each study was also calculated (Fig. 2A-F).

### Clinical significance of KIAA1429 in OS

**Figure 3:**
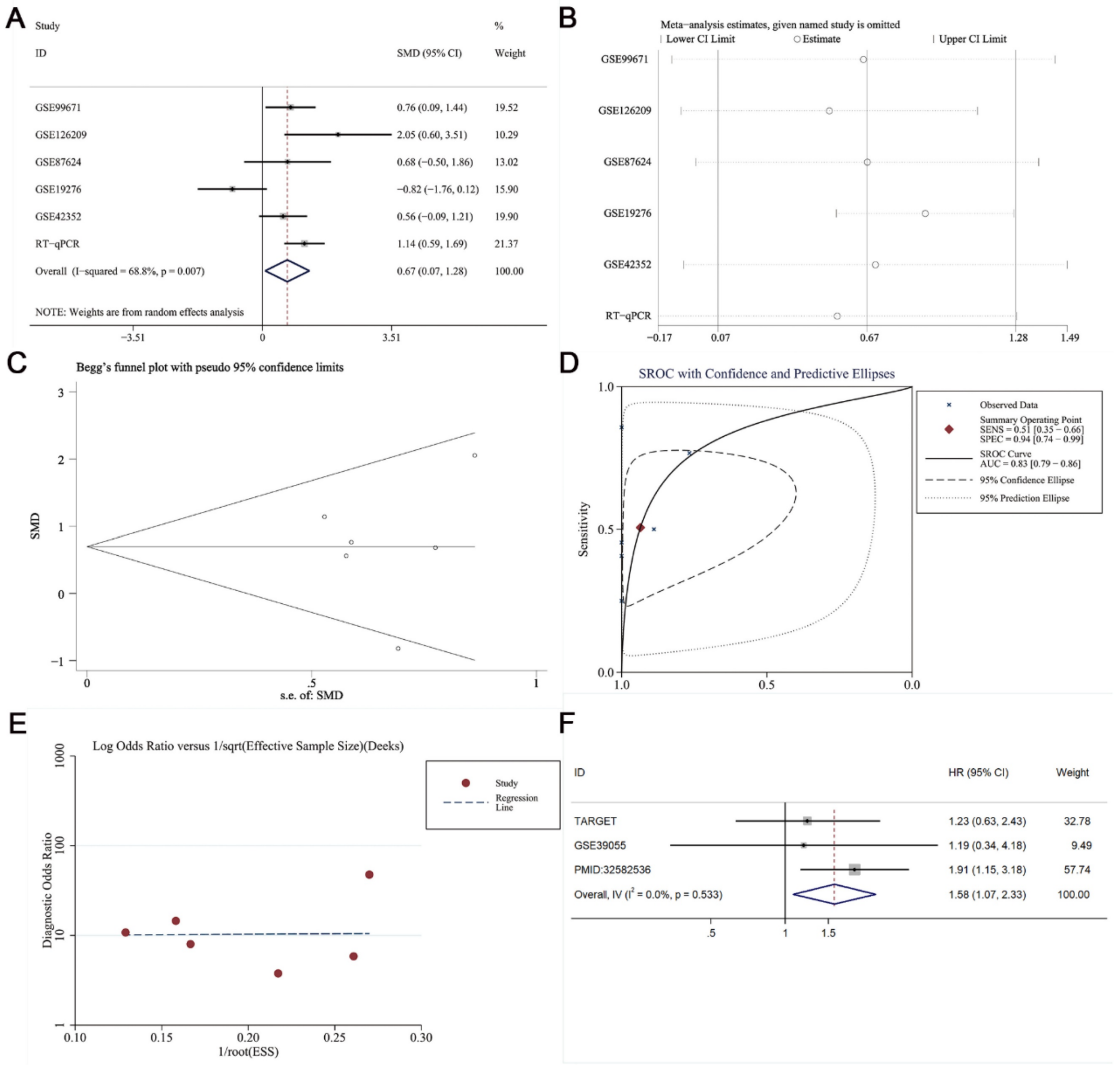
** Clinical significance of KIAA1429 in OS was evaluated by meta-analysis.** (A) Forest diagram of KIAA1429 expression in OS. (B) Sensitivity analysis of KIAA1429 expression in OS. (C) Begg's funnel diagram, which suggested no publication bias. (D) The sROC curve showed that KIAA1429 had a good capability to discriminate OS samples from non-cancer samples. (E) Deek's funnel diagram, which suggested no publication bias. (F) Forest plot of the effects of KIAA1429 expression on disease prognosis in OS. OS: osteosarcoma.

RT-qPCR, microarray, and RNA sequencing data were acquired for a total of 250 OS samples and 71 non-cancer samples (Table 1). KIAA1429 expression was notably increased in OS samples than non-cancer samples, as indicated by the standard mean difference (SMD = 0.67, 95% CI 0.07-1.28) obtained from the random effects model (Fig. 3A). Sensitivity analysis showed that no studies had a significant impact on the entire study cohort (Fig. 3B); Begg's test and Egger's test results suggested that no publication bias was observed (*p* > 0.05) (Fig. 3C). The area under the curve (AUC) value of the summary receiver operating characteristic (sROC) curve was 0.83, showing that KIAA1429 demonstrated good diagnostic capacity to distinguish OS samples from non-cancer samples (Fig. 3D), and no publication bias was observed (Fig. 3E). The relationship between KIAA1429 expression and clinical features in patients with OS was analyzed using data from the TARGET database. Higher expression of KIAA1429 was significantly associated with disease recurrence, but not with gender, age, primary location, disease metastasis, or survival status (Table 2). Survival analysis results showed that patients with high KIAA1429 expression had a shorter overall survival time [HR, 1.58 (1.07, 2.33)] (Fig. 3F).

### Silencing KIAA1429 reduces m6A methylation levels, inhibits cell proliferation, prevented the growth of tumors *in vivo* and promotes apoptosis in OS cells

**Figure 4:**
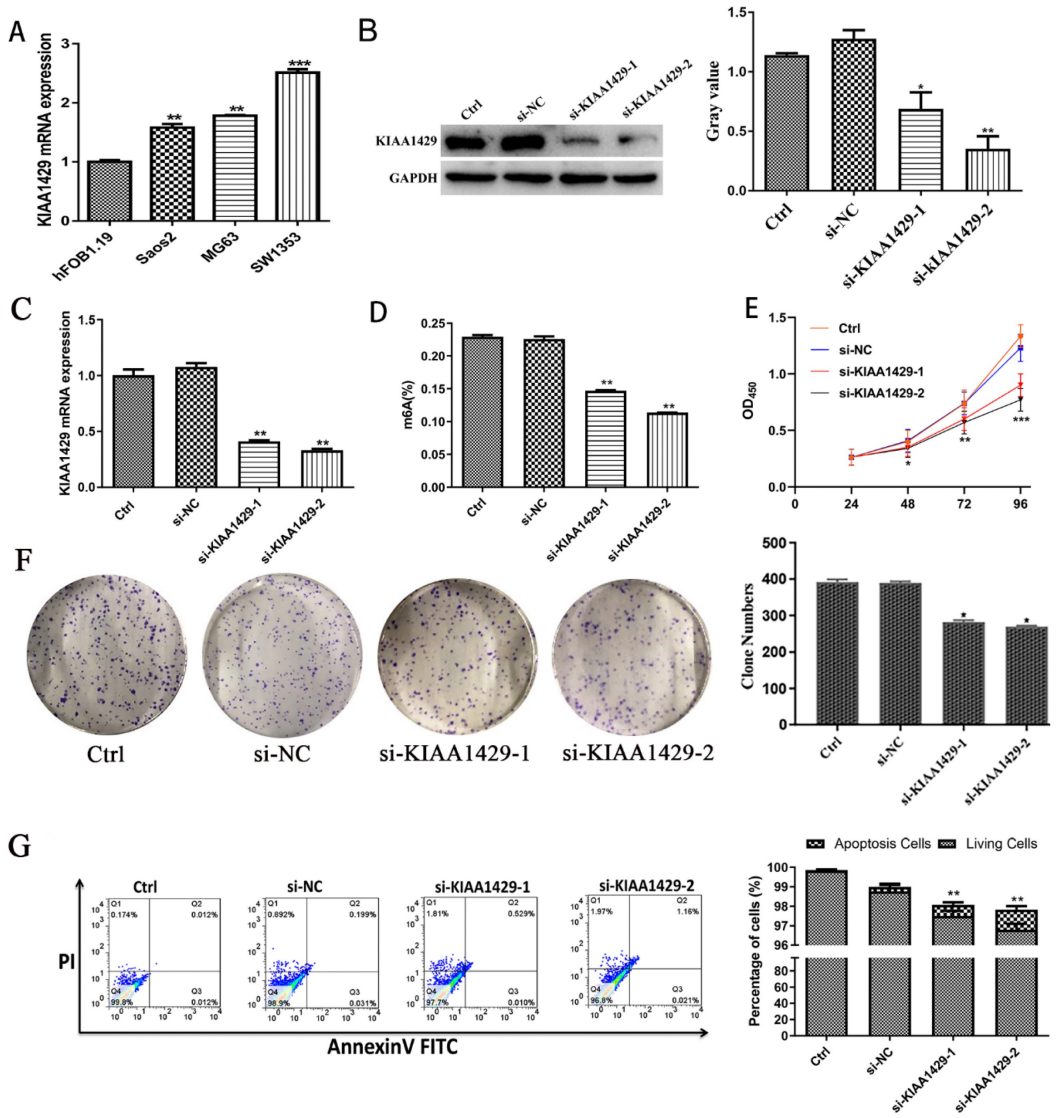
**Biological function of KIAA1429 in OS.** (A) The expression of KIAA1429 in OS cells and osteoblasts. (B) The knockdown efficiency of KIAA1429 in SW1353 cells was confirmed by western blot. (C) The knockdown efficiency of KIAA1429 in SW1353 cells was confirmed by RT-qPCR. (D) EpiQuik m6A RNA Methylation Quantification Kit was used to evaluate the content of m6A in SW1353 cells after silencing KIAA1429. (E) Cell counting kit-8 assay was performed to assess KIAA1429-knockdown on cell proliferation in SW1353 cells. (F) Silencing KIAA1429 impaired colony forming ability in SW1353 cells. (G) Flow cytometry assay was applied to determine the effects of KIAA1429-knockdown on cell apoptosis in SW1353 cells. OS: osteosarcoma. *p <0.05; **p <0.01.

The expression of KIAA1429 in OS cells was notably increased than in osteoblast cells (Fig. 4A). To explore the function of KIAA1429 in OS, we knocked down the expression of KIAA1429 in the SW1353 cell line, which had the highest expression of KIAA1429 among three OS cell lines we assessed. RT-qPCR and western blot analysis demonstrated that both siRNA constructs, si-KIAA1429-1 and si-KIAA1429-2, exhibited good KIAA1429 knockdown efficiency (Fig. 4B-C). Silencing KIAA1429 reduced m6A methylation levels in SW1353 cells (Fig. 4D). Knocking down KIAA1429 significantly inhibited the proliferation SW1353 cells, as determined by CCK-8 assays (Fig. 4E). In comparison with the si-NC group, silencing KIAA1429 was found to efficiently suppress colony formation ability of SW1353 cells, suggesting that KIAA1429 promotes the proliferation of OS cells (Fig. 4F). Apoptotic cells in the both si-KIAA1429-1 and si-KIAA1429-2 treated groups were significantly increased compared with the si-NC group (Fig. 4G).

**Figure 5:**
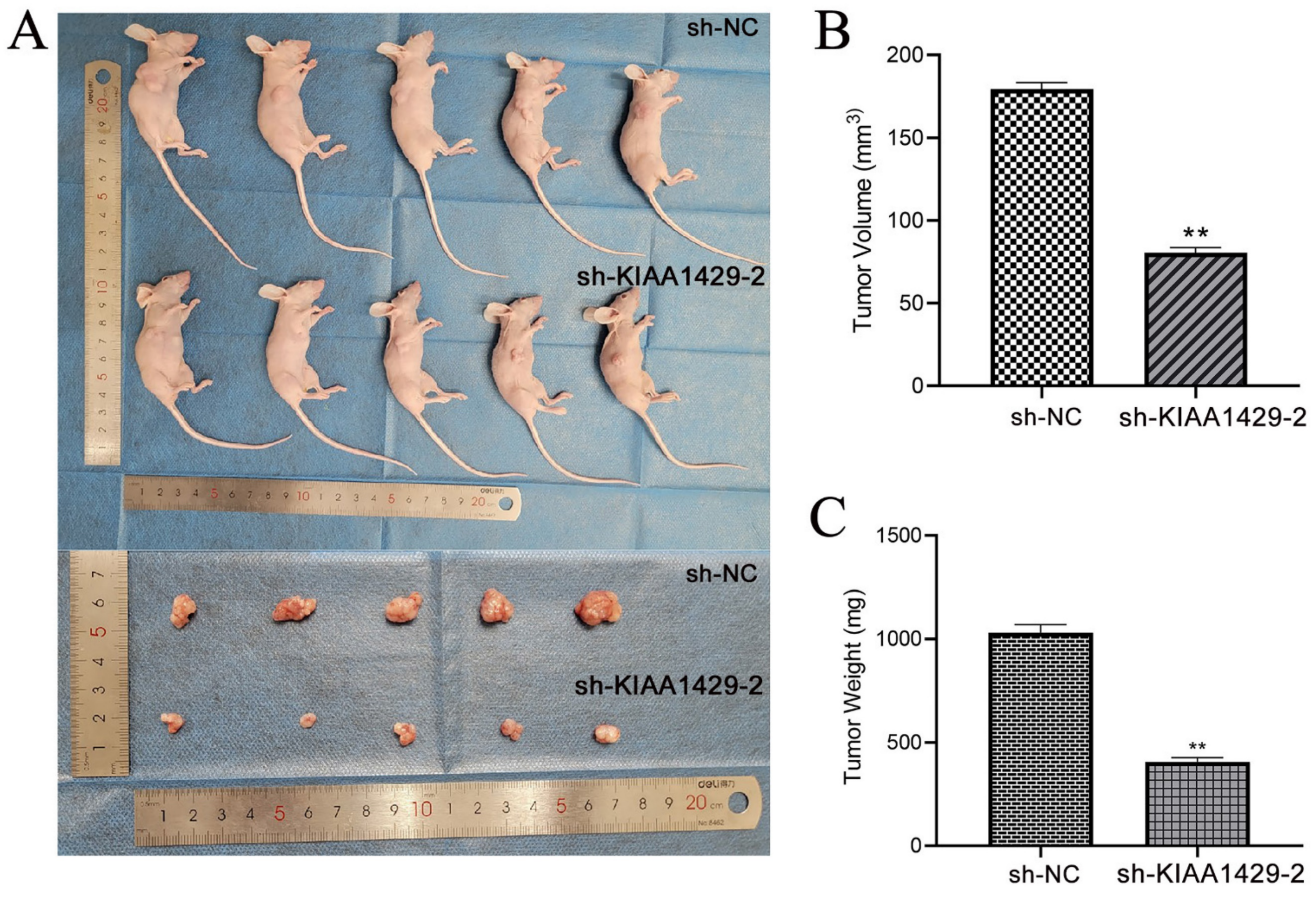
** OS cells tumorigenicity diminishes *in vivo* when KIAA1429 was knocked down**. (A) The size of xenograft tumors *in vivo* markedly declined after KIAA1429 was knockdown. (B) After silencing KIAA1429, the OS cells transplanted into naked mice have a significantly lower volume. (C) After silencing KIAA1429, the OS cells transplanted into naked mice have a significantly lower weight. OS: osteosarcoma. *p <0.05; **p <0.01.

Nude mice were injected with SW1353 cells transfected with sh-NC or sh-KIAA1429-2 plasmids below the right axilla, and tumor progression following cell transplantation were assessed. The findings revealed that the SW1353 cells' tumor-promoting properties were clearly inhibited by *in vivo* silencing of KIAA1429. This was demonstrated by the size and weight of tumors in the NC group, as they were noticeably larger than those in the sh-KIAA1429-2 group (Fig. 5A-C).

### Bioinformatics analysis of genes related to KIAA1429

**Figure 6:**
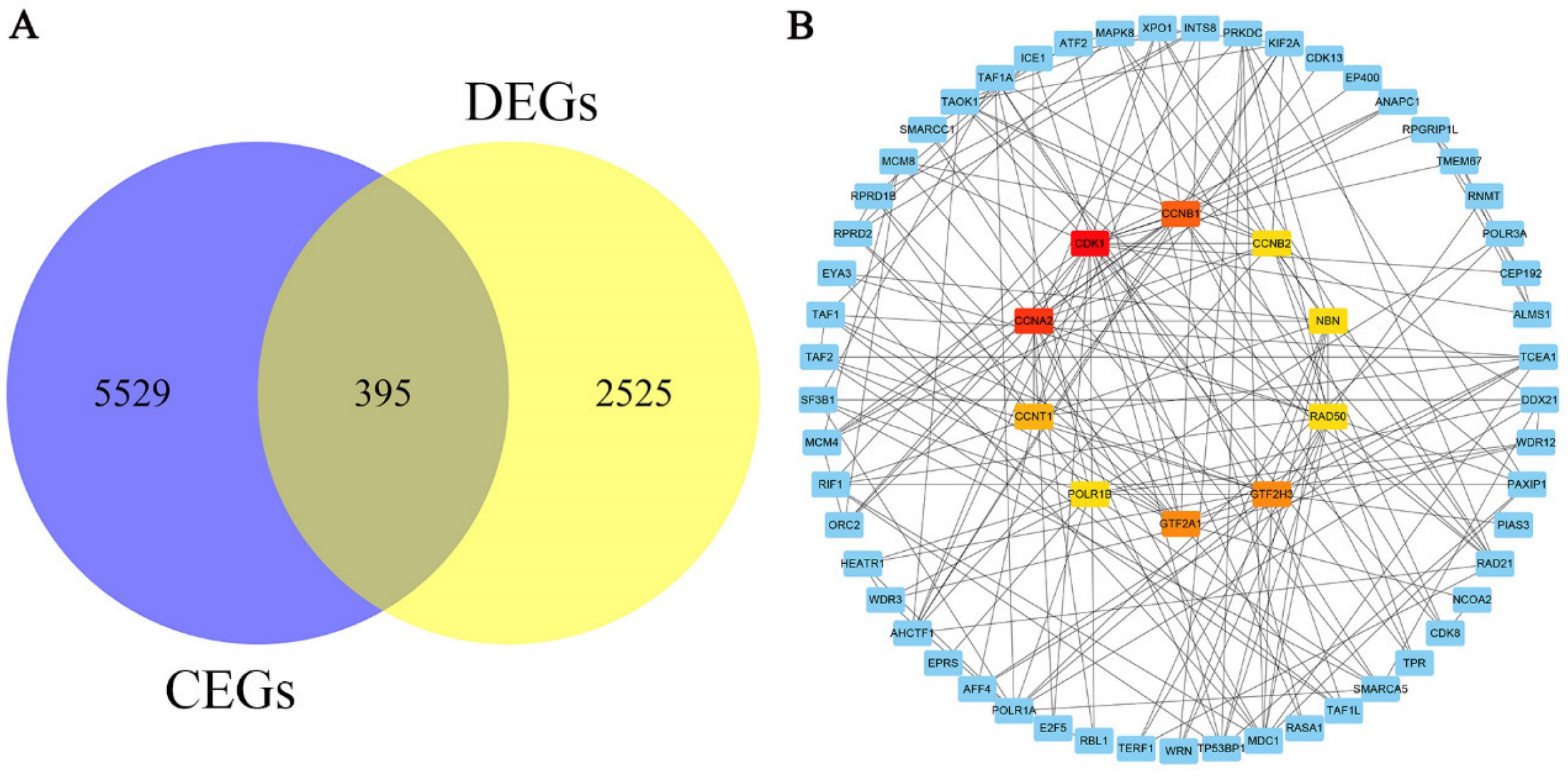
** The screening and PPI network analysis of KIAA1429-related genes.** (A) A total of 395 KIAA1429-related genes were identified from CEGs and DEGs in OS. (B) PPI network analysis showed that CDK1, CCNA2, and CCNB1 had the top three degrees of connection of the KIAA1429 related genes. CEGs: co-expressed genes; DEGs: differentially expressed genes; OS: osteosarcoma.

**Figure 7:**
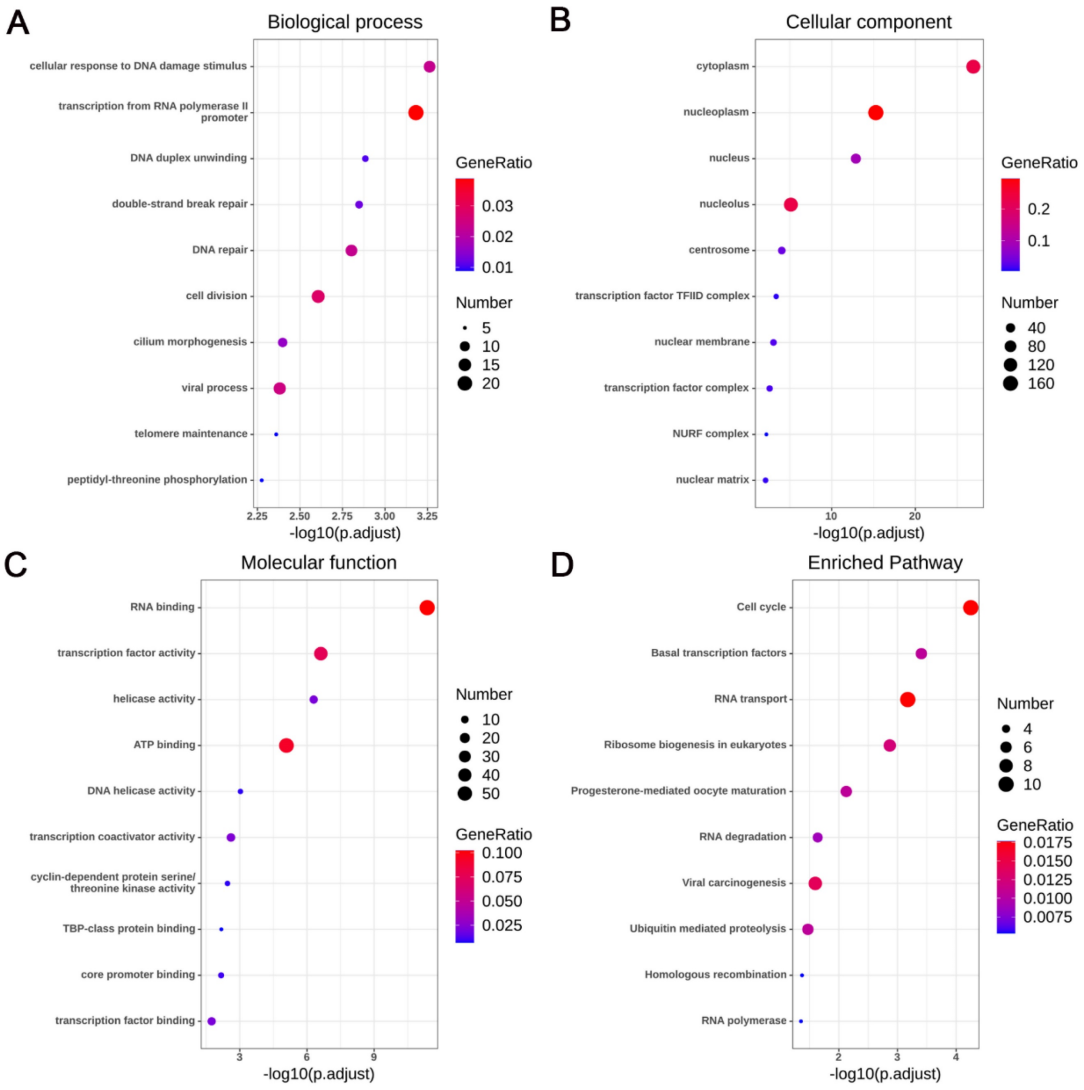
** Functional and pathway enrichment analyses of the KIAA1429-related genes.** (A) Biological process. (B) Cellular component. (C) Molecular function. (D) KEGG pathways.

A total of 5924 CEGs and 2920 DEGs related to KIAA1429 were identified in OS; 395 genes represented the intersection between both gene lists, and were collectively termed the KIAA1429-related genes (Fig. 6A). Three genes, CDK1, CCNA2, and CCNB1, were screened as hub related genes in OS using PPI network analysis (Fig. 6B). GO enrichment analysis of the 395 KIAA1429-related genes demonstrated that these genes were enriched most significantly in cellular response to DNA damage stimulus, nucleoplasm, and RNA binding (Table 3) (Fig. 7A-C). KEGG pathway analysis results suggested that the cell cycle pathway was most significantly enriched pathway among the KIAA1429-related genes (Table 4) (Fig. 7D).

### Validation of hub related genes

By integrating microarray and RNA sequencing datasets (Table 5), we found that CDK1, CCNA2, and CCNB1 were significantly upregulated in OS samples than non-cancer samples (Supplementary Fig. 2A, 2C, and 2D). The summary receiver operating characteristic (SROC) curve analysis shows that CDK1, CCNA2, and CCNB1 have a good capability to distinguish between OS samples and non-OS samples (Supplementary Fig. 2B, 2D, and 2F). Survival analysis showed that overexpression of CCNA2 was associated with shorter overall survival time, and that expression of CDK1 and CCNB1 was not associated with overall survival outcomes in OS (Supplementary Fig. 3).

We identified high expression and moderate diagnostic capacity of KIAA1429 in 321 OS samples; high KIAA1429 expression was associated with poor prognosis. Cell function experiments validated that knocking down KIAA1429 reduced the m6A methylation level of OS cells, inhibited their proliferation, and accelerated OS cell apoptosis. Functional enrichment analysis was performed to elucidate the potential molecular mechanism of KIAA1429. PPI network analysis showed the highest connectivity among CDK1, CCNA2, and CCNB1. On integrating microarray and RNA-seq datasets, it was confirmed that CDK1, CCNA2, and CCNB1 expression levels were upregulated in OS, indicative of their promising diagnostic potential. Thus, we speculate that KIAA1429 promotes OS progression by targeting CDK1, CCNA2, and CCNB1.

## Discussion

KIAA1429, a member of the RNA methyltransferase complex, is involved in the tumorigenesis and development of a variety of cancers. Lan *et al.* reported that KIAA1429 promotes the progression of liver cancer by reducing GATA3 expression through an m6A-dependent pathway 18. Cheng *et al.* found that KIAA1429 upregulates the m6A modification levels of ID2 mRNA, leading to inhibition of ID2 mRNA expression and promoting the metastasis of liver cancer 19. In breast cancer, KIAA1429 was shown to upregulate the expression of CDK1, thereby promoting the proliferation of breast cancer cells 20. KIAA1429 is also involved in the progression of gastric cancer, where it upregulates the expression of c-Jun in an m6A-independent manner 21. The only notable study of KIAA1429 in OS reported that KIAA1429 expression was higher in OS lesions compared to matched non-cancerous tissue 22. However, that study involved a small sample size (n = 30), and the observations required confirmation in a larger study including many more OS samples. In the present study, we analyzed 321 samples from six studies, and we found that KIAA1429 expression is significantly elevated in OS. Moreover, we observed that overexpression of KIAA1429 is related to poorer disease prognosis for patients with OS. Furthermore, we demonstrated that silencing KIAA1429 reduced m6A methylation levels, inhibited proliferation, and promoted apoptosis in OS cells. These results suggested that KIAA1429 plays a role as an oncogene in OS, and is a potential prognostic biomarker of poorer clinical outcomes.

To further explore the potential biological mechanisms by which KIAA1429 facilitates OS, we performed enrichment analysis of KIAA1429-related genes (obtained by merging the intersection of CEGs and DEGs). We found that KIAA1429-related genes were significantly enriched in the cell cycle pathway. Multiple studies have shown that KIAA1429-related genes. are involved in the progression of cancer by regulating the cell cycle pathway. For example, the lncRNA PITPNA-AS1/miR-876-5p/c-MET axis regulates the cell cycle, and promotes the progression of cervical cancer 36. Additionally, HE4 overexpression reduces the sensitivity of pancreatic cancer to paclitaxel by deregulating the cell cycle pathway 37. Glycyrrhizic acid represses the proliferation of gastric cancer cells by inhibiting the cell cycle 38. High expression of FOXF1 inhibits the progression of lung cancer cells by inducing G1 cell cycle arrest 39. Silencing of REG γ mediates apoptosis of OS cells by inhibiting the cell cycle 40. Furthermore, miRNA-98-5p regulates the cell cycle in OS by downregulating the expression of CDC25A, which in turn inhibits the progression of OS 41. We hypothesize that KIAA1429 is involved in OS progression through regulation of the cell cycle pathway. However, further experiments are needed to verify the relationship between KIAA1429 and regulation of the cell cycle.

In the present study, three hub related genes, CDK1, CCNA2, and CCNB1, were identified by PPI network analysis, and were shown to play important roles in the development of OS. Yang *et al*. found that CDK1 expression might be relate to methotrexate resistance in OS 42. FGFR1 was shown to upregulate the expression of CDK1 to promote the proliferation of OS cells 43. Piperine inhibited the progression of OS by reducing the expression of CDK1 44. Ginsenosides induced apoptosis and cell cycle arrest of OS cells by reducing CDK1 expression 45. PDCD5 downregulated the expression of CDK1, which induced apoptosis in MG-63 cells 46. In this study, by merging microarray and RNA sequencing data, we found that CDK1 was significantly overexpressed in OS, and had the capability to distinguish between OS and non-OS, as demonstrated by summary receiver operating characteristic (sROC) analysis. By analyzing 419 samples collected from microarray and sequencing datasets, we found that the expression of CCNA2 is upregulated in OS, and that CCNA2 is a good candidate for a diagnostic biomarker in OS. Liu *et al.* showed that knockdown of CCNA2 remarkably inhibited proliferation of OS cells 47. The regulation of CCNA2 by miR-449a and miR-424 has been reported to be involved in the progression of OS 48. Additionally, Wu *et al.* found that CCNA2 expression is significantly upregulated in OS cell lines, and reported that patients with high expression of CCNA2 had worse prognosis 49; this is consistent with our finding that elevated CCNA2 was associated with poorer clinical outcome.

CCNB1, a cell cycle correlated gene, may play a key role in the tumorigenesis and progression of OS 50. FKBP14 increased the number of OS cells in G0/G1 phase by regulating the expression of CCNB1 51. In a study that evaluated 5 OS samples and 22 normal tissue samples, Bekim *et al*. showed that there was no difference of CCNB1 expression between OS tissue and normal tissue 52. Wang *et al*. reported that CCNB1 is significantly downregulated in OS tissue compared with normal tissue, but their study evaluated just 14 OS samples and 3 normal tissue samples 53. In the present study, we found that CCNB1 was upregulated in 321 OS samples than 66 non-cancer samples; the larger sample size that we used in our analysis gives our study stronger statistical power compared to the small sample sizes in prior studies.

Of course, this study has certain limitations. First, we only tested the mRNA level of KIAA1429 in OS, and a further immunohistochemistry experiment is needed to evaluate the expression of KIAA1429 at the protein level. Second, the heterogeneity of this study was 68.8%, which weakened the credibility of our results to a certain extent. Due to insufficient sample information, we cannot perform subgroup analysis or meta-regression to find the source of heterogeneity, and a random effects model was applied. Third, our results showed that KIAA1429 plays an oncogenic role in the progression of OS, but its underlying regulation mechanism needs to be further investigated. Fourth, the relationship among CDK1, CCNB1, CCNA2, and KIAA1429 and the underlying regulatory mechanisms need to be explored via *in vivo* and *in vitro* experiments. Finally, future studies are warranted to investigate changes in m6A methylation levels of CDK1, CCNB1, and CCNA2 and to assess whether KIAA1429 regulates the methylation levels of these genes.

In conclusion, KIAA1429 may contribute to the progression of OS by targeting CDK1, CCNA2, and CCNB2. KIAA1429 could serve as a potential biomarker and therapeutic target in OS.

## Conclusion

In summary, our results suggest that KIAA1429 is significantly overexpressed in osteosarcoma samples and has moderate diagnostic capacity (standardized mean difference=0.67, area under the curve=0.83). KIAA1429 may contribute to the progression of osteosarcoma by targeting CDK1, CCNA2, and CCNB2, which make it possible to become a potential biomarker and therapeutic target of osteosarcoma.

## Supplementary Material

Supplementary figures and table.

## Figures and Tables

**Table 1:** The KIAA1429 expression in OS samples and non-OS samples based on RT-qPCR, microarray and RNA sequencing data.

Study	Country	Year	Platform	Non-OS			OS		
				N	M	SD	N	M	SD
GSE99671	Estonia	2017	GPL20148	18	9.389	0.223	18	9.642	0.411
GSE126209	China	2019	GPL20301	5	3.039	0.081	6	3.475	0.266
GSE87624	USA	2016	GPL11154	3	3.513	0.276	44	3.847	0.498
GSE19276	Australia	2009	GPL6848	5	0.588	0.000	44	0.023	0.720
GSE42352	Norway	2012	GPL10295	10	9.288	0.162	108	9.575	0.381
RT-qPCR	China	2019	--	30	0.521	0.405	30	1.447	0.999

M: mean; N: number; OS: osteosarcoma; SD: standard deviation.

**Table 2:** Association between KIAA1429 expression and clinicopathological parameters in OS samples based on TARGET database.

Clinicopathological		KIAA1429 expression		t-test	
parameters	N	M	SD	t value	*p-*value
Gender					
Female	37	11.511	0.898	-1.447	0.152
Male	48	11.790	0.869		
Age					
<18	67	11.598	0.825	-1.413	0.161
≥18	18	11.929	1.074		
Primary location					
Femur/Tibia	61	11.619	0.904	-0.824	0.412
Others	24	11.795	0.849		
Recurrence					
No	45	11.353	1.017	-2.884	0.005^*^
Yes	40	11.873	0.617		
Metastasis					
No	63	11.760	0.892	1.62	0.109
Yes	22	11.407	0.840		
Survival state					
Alive	58	11.713	0.925	0.673	0.503
Dead	27	11.573	0.809		

TARGET: Therapeutically Applicable Research to Generate Effective Treatments; M: mean; N: number; OS: osteosarcoma; SD: standard deviation.

**Table 3:** The 10 most significant items of the GO analyses based on 395 KIAA1429-related genes.

Category	Term	Count	*p-*value
GOTERM_BP_DIRECT	cellular response to DNA damage stimulus	13	5.47E-04
GOTERM_BP_DIRECT	transcription from RNA polymerase II promoter	22	6.58E-04
GOTERM_BP_DIRECT	DNA duplex unwinding	6	1.31E-03
GOTERM_BP_DIRECT	double-strand break repair	7	1.42E-03
GOTERM_BP_DIRECT	DNA repair	13	1.58E-03
GOTERM_BP_DIRECT	cell division	16	2.47E-03
GOTERM_BP_DIRECT	cilium morphogenesis	9	4.00E-03
GOTERM_BP_DIRECT	viral process	14	4.16E-03
GOTERM_BP_DIRECT	telomere maintenance	5	4.36E-03
GOTERM_BP_DIRECT	peptidyl-threonine phosphorylation	5	5.31E-03
GOTERM_CC_DIRECT	nucleoplasm	131	1.21E-27
GOTERM_CC_DIRECT	nucleus	167	5.34E-16
GOTERM_CC_DIRECT	nucleolus	51	1.28E-13
GOTERM_CC_DIRECT	cytoplasm	131	7.41E-06
GOTERM_CC_DIRECT	centrosome	21	9.04E-05
GOTERM_CC_DIRECT	transcription factor TFIID complex	6	4.25E-04
GOTERM_CC_DIRECT	nuclear membrane	13	8.96E-04
GOTERM_CC_DIRECT	transcription factor complex	11	2.56E-03
GOTERM_CC_DIRECT	NURF complex	3	6.31E-03
GOTERM_CC_DIRECT	nuclear matrix	7	7.93E-03
GOTERM_MF_DIRECT	RNA binding	58	4.28E-12
GOTERM_MF_DIRECT GOTERM_MF_DIRECT	transcription factor activity	43	2.40E-07
	helicase activity	12	5.01E-07
GOTERM_MF_DIRECT GOTERM_MF_DIRECT GOTERM_MF_DIRECT	ATP binding DNA helicase activity	53 5	8.28E-06 9.41E-04
	transcription coactivator activity	13	2.49E-03
GOTERM_MF_DIRECT	cyclin-dependent protein serine/threonine kinase activity	5	3.55E-03
GOTERM_MF_DIRECT	TBP-class protein binding	4	6.67E-03
GOTERM_MF_DIRECT	core promoter binding	6	6.80E-03
GOTERM_MF_DIRECT	transcription factor binding	12	1.80E-02

GO: gene ontology; BP: biological process; CC: cellular component; MF: molecular function.

**Table 4:** The 10 most significant items of the KEGG analyses based on 395 KIAA1429-related genes.

Category	Term	Count	*p*-value
KEGG_PATHWAY	Cell cycle	10	5.65E-05
KEGG_PATHWAY	Basal transcription factors	6	3.93E-04
KEGG_PATHWAY	RNA transport	10	6.70E-04
KEGG_PATHWAY	Ribosome biogenesis in eukaryotes	7	1.36E-03
KEGG_PATHWAY	Progesterone-mediated oocyte maturation	6	7.47E-03
KEGG_PATHWAY	RNA degradation	5	2.30E-02
KEGG_PATHWAY	Viral carcinogenesis	8	2.53E-02
KEGG_PATHWAY	Ubiquitin mediated proteolysis	6	3.37E-02
KEGG_PATHWAY	Homologous recombination	3	4.25E-02
KEGG_PATHWAY	RNA polymerase	3	4.43E-02

KEGG: Kyoto Encyclopedia of Genes and Genomes.

**Table 5:** The descriptions of selected microarray and RNA sequencing datasets.

Study	Year	Country	Platform	OS samples	Non-OS samples
E-MEXP-3628	2012	USA	NA	4	14
GSE12865	2009	Canada	GPL6244	12	2
GSE126209	2019	China	GPL20301	6	5
GSE99671	2017	Estonia	GPL20148	18	18
GSE87624	2016	USA	GPL11154	44	3
GSE68591	2015	USA	GPL11028	10	2
GSE39262	2012	UK	GPL96	10	3
GSE14359	2010	Germany	GPL96	18	2
GSE11414	2009	Canada	GPL6244	4	2
GSE42352	2012	USA	GPL10295	108	10
GSE36001	2012	Norway	GPL6102	19	6
GSE33383	2011	Norway	GPL10295	84	15

OS: osteosarcoma.

## Abbreviations

OS: osteosarcoma
CEGs: co-expressed genes
DEGs: differentially expressed genes
PPI: Protein-protein interaction
m6A: N6-methyladenosine
SRA: Sequence Read Archive
TARGET: Therapeutically Applicable Research to Generate Effective Treatments
CCK-8: Cell counting kit-8
PI: propidium iodide
DAVID: Database for Annotation, Visualization, and Integrated Discovery
GO: Gene Ontology
KEGG: functions and Kyoto Encyclopedia of Genes and Genomes
STRING: Search Tool for the Retrieval of Interacting Genes
sROC: summarized receiver operating characteristic
ROC: Receiver operating characteristic
SMD: standardized mean difference
HR: hazard radio
95%CI: 95% confidence interval
AUC: area under the curve
