# Supplementary material for: Clinical Significance and Potential Mechanisms of the RNA Methyltransferase KIAA1429 in Osteosarcoma
Source: J Cancer. 2024 Jan 1;15(1):126–39. doi: 10.7150/jca.86630 (PMC10751680; doi:10.7150/jca.86630)
Supplement: Supplementary file 1 — Supplementary figures and table. [file jcav15p0126s1.pdf]

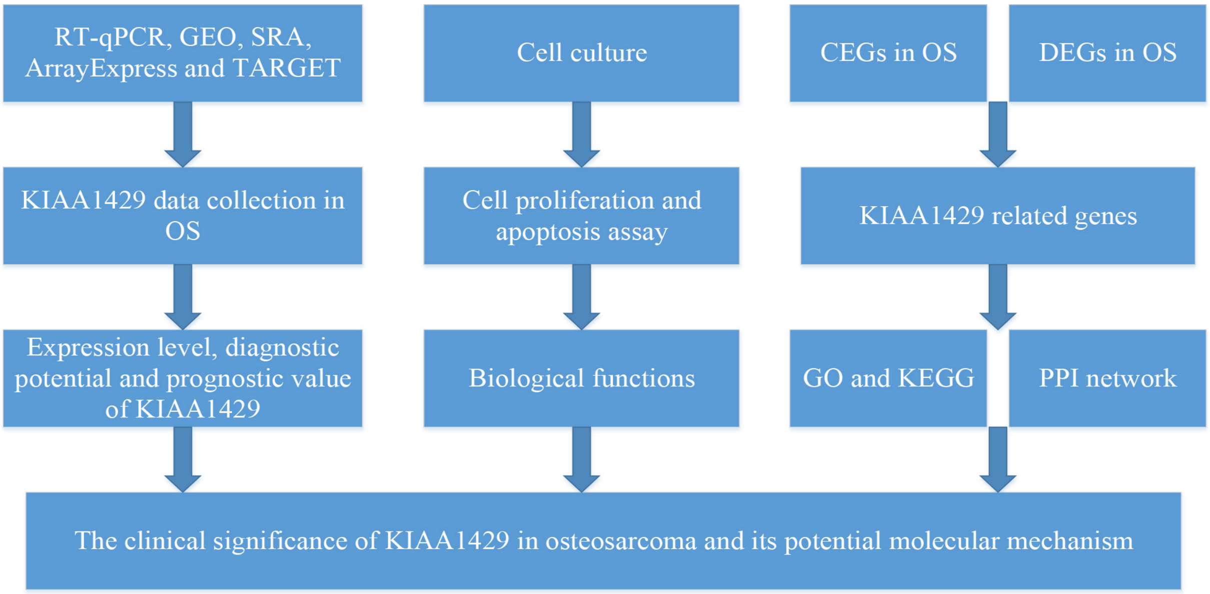

Supplement Fig. 1. Flow diagram of the study procedure. GEO: Gene Expression Omnibus; SRA: Sequence Read Archive; TARGET: Therapeutically Applicable Research To Generate Effective Treatments; CEGs: co-expressed genes; DEGs: differentially expressed genes; GO: Gene Ontology; KEGG: Kyoto Encyclopedia of Genes and Genomes; PPI: protein–protein interaction.

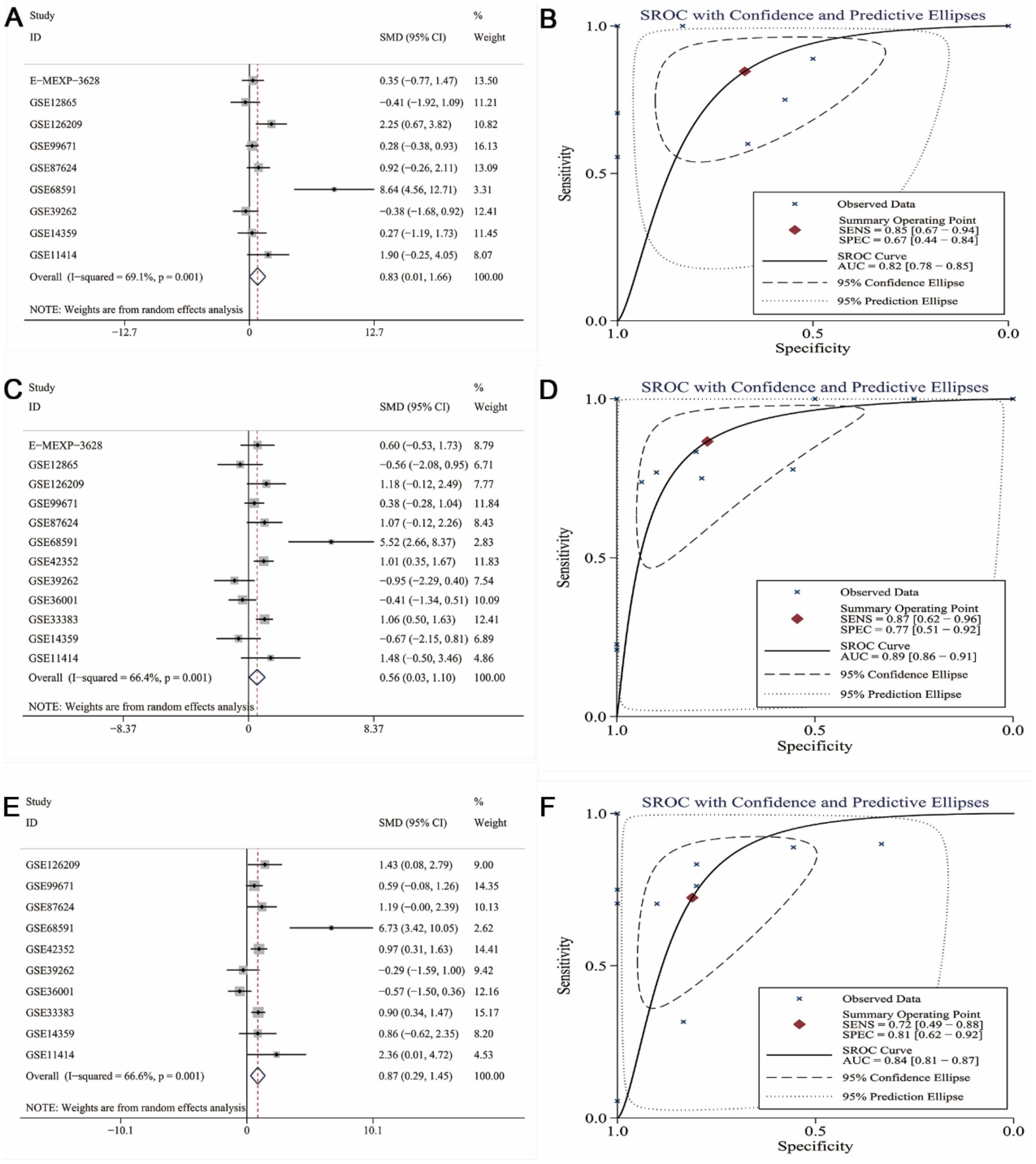

Supplement Fig. 2. The expression and diagnostic capability of three hub related genes (CDK1, CCNA2 and CCNB1) in OS based on microarray and RNA sequencing data. (A) CDK1 was overexpressed in OS samples compared with control samples. (B) CDK1 demonstrated strong diagnostic potential between OS and control samples. (C) Overexpressed CCNA2 in OS samples compared with control samples. (D) CCNA2 demonstrated strong diagnostic potential between OS and control samples. (E) Overexpressed CCNB1 in OS samples compared with control samples. (F) CCNB1 demonstrated strong diagnostic potential between OS and control samples.

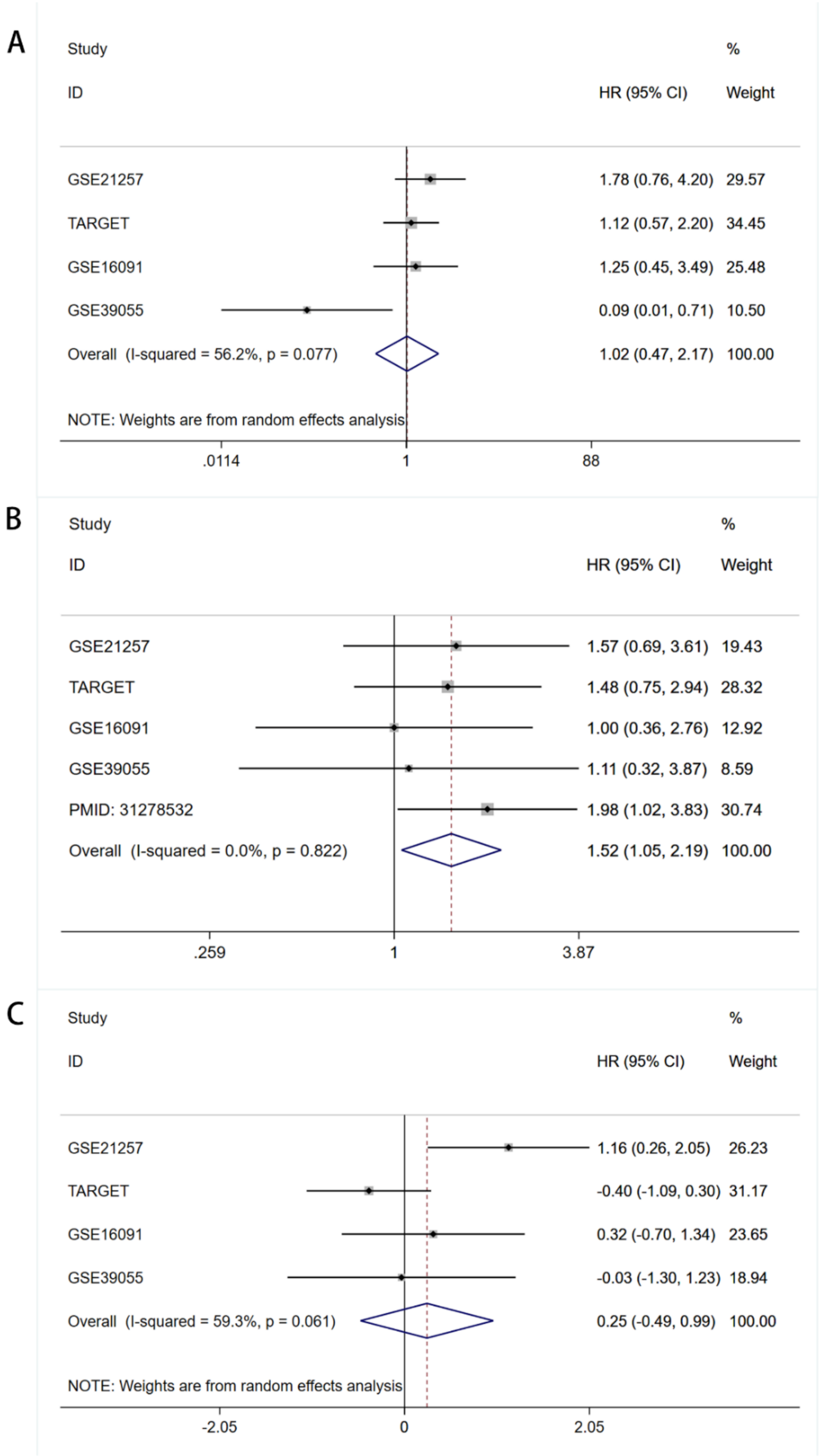

Supplement Fig. 3. Survival analysis of three hub related genes (CDK1, CCNA2 and CCNB1) in OS based on microarray, RNA sequencing, and published data. (A) CDK1 expression was not associated with overall survival of OS patients. (B) Overexpression of CCNA2 associated with poorer prognosis among OS patients. (C) CCNB1 expression was not associated with overall survival of OS patients.

Supplement Table 1. Clinical parameter of OS patients in this study.

Patient Number	gender	age	lesion location
1	male	22	Lower femur
2	female	12	Upper fibula
3	male	17	Upper fibula
4	female	12	Upper tibia
5	male	10	Lower femur
6	male	12	Lower femur
7	female	9	Upper fibula
8	female	13	Lower femur
9	female	15	Upper tibia
10	male	9	Lower femur
11	female	10	Lower femur
12	female	13	Lower femur
13	male	30	Upper fibula
14	male	14	Upper tibia
15	male	29	Lower femur
16	female	15	Lower femur
17	male	14	Lower femur
18	female	12	Lower femur
19	male	14	Lower femur
20	male	31	Upper tibia
21	female	10	Upper tibia
22	male	11	Lower femur
23	male	12	Lower femur

24	female	14	Lower femur
25	female	9	Upper tibia
26	male	16	Lower femur
27	male	11	Lower femur
28	male	13	Lower femur
29	female	21	Upper tibia
30	female	19	Upper fibula
